# Tryptophan-Assisted Synthesis Reduces Bimetallic Gold/Silver Nanoparticle Cytotoxicity and Improves Biological Activity

**DOI:** 10.5772/59684

**Published:** 2014-01-01

**Authors:** Igor O. Shmarakov, Iuliia P. Mukha, Volodymyr V. Karavan, Olexander Yu. Chunikhin, Mykhailo M. Marchenko, Natalia P. Smirnova, Anna M. Eremenko

**Affiliations:** 1 Department of Biochemistry and Biotechnology, Chernivtsi National University, Chernivtsi, Ukraine; 2 Laboratory of Surface Photonics, Institute of Surface Chemistry, NAS of Ukraine, Kyiv, Ukraine; 3 A. V. Palladin Institute of Biochemistry, NAS of Ukraine, Kyiv, Ukraine

**Keywords:** gold, silver, nanoparticles, tryptophan-assisted synthesis, hepatotoxicity, anticancer activity

## Abstract

Aiming to reduce the potential in vivo hepato-and nephrotoxicity of Ag/Au bimetallic nanoparticles (NPs) stabilized by sodium dodecyl sulphate (SDS), an approach involving a simultaneous reduction of silver nitrate and tetrachlorauratic acid using tryptophan (Trp) as a reducing/stabilizing agent was applied during NP synthesis. The obtained Ag/Au/Trp NPs (5–15 nm sized) were able to form stable aggregates with an average size of 370–450 nm and were potentially less toxic than Ag/Au/SDS in relation to a mouse model system based on clinical biochemical parameters and oxidative damage product estimation. Ag/Au/Trp NPs were shown to exhibit anticancer activity in relation to a Lewis lung carcinoma model. The data generated from the present study support the fact that the use of tryptophan in NP synthesis is effective in attenuating the potential hepatotoxicity and nephrotoxicity of NPs during their in vivo application.

## 1. Introduction

In recent years, the use of nanomaterials, particularly metal nanoparticles (NPs), has expanded in biomedical research. They are used in diagnosis and therapeutics due to their unique properties, including small size, large surface area-to-volume ratio, high reactivity to living cells, stability under high temperatures, and translocation into the cells [1]. Enormous scientific and technological interest has been given to gold and silver NPs because of their easy synthesis, chemical stability and unique properties. The intensive use of nanotechnology in modern biological science, including chemical sensing, biological imaging, drug delivery, cancer treatment and considerable advances in their application [1, 2], do not reject the question of NP's adverse effects in relation to biological systems [3]. Due to their nanoscale dimensions, they exhibit qualitatively new properties which underlie their biological activity and possible toxic effects. In spite of their extraordinary capacity for bioconjugation with various molecules, there are studies showing NPs to be cytotoxic due to their inherent physico-chemical properties. Special attention should be paid to the potential hepato-and nephrotoxic effects of NPs at the organism level [4, 5], since the liver is the main homeostatic organ and is the leading site of xenobiotic detoxication in the body. The liver has the highest ability to accumulate NPs and is the main target organ for NPs that are administered, which greatly increases the risk of their toxic effects. A general mechanism of nanomaterial toxicity is an induction of oxidative stress in cells, where the molecular targets of colloidal metal NPs are the lipid components of the plasma and intracellular membranes and associated protein complexes [6]. Nonspecific oxidative damage is one of the greatest concerns limiting the implementation of NPs, and the approaches aiming to attenuate this phenomenon appear to be very important.

In this study, we estimated the potential *in vivo* toxicity of colloidal Ag/Au NPs stabilized by sodium dodecyl sulphate (SDS) and by the essential amino acid tryptophan (Trp), with the specific aim of the possible improvement of NPs biocompatibility using a natural compound as a surface-bound stabilizing ligand. Finally, the anti-tumour activity, as one of the possible application of NPs, was also studied.

## 2. Experimental procedures

### 2.1 Synthesis and characterization of NPs

To obtain Ag/Au NPs, the following materials were used: silver nitrate, tetrachlorauratic acid (AgNO_3_, HAuCl_4_, Merck, Germany), sodium dodecyl sulphate (SDS, Sigma Aldrich, Germany), sodium tetrahydroborate (NaBH_4_, Fluka, Germany) and tryptophan (Trp, SC12–20120713, China). Ag/Au NPs in a colloidal solution were obtained in two ways. 1. The simultaneous chemical reduction of silver nitrate and tetrachlorauratic acid in the presence of sodium tetrahydroborate using as stabilizer surfactant SDS in a molar ratio to the metal of 3:1. The concentrations in the resulting solution used were C(M)=3 10^−3^M (C_Ag_=C_Au_=1,5 10^−3^ M), C(NaBH_4_)=9 10^−3^ M. 2. To reduce the toxicity of the NPs, the simultaneous reduction of silver nitrate and tetrachlorauratic acid using tryptophan as a reducing/stabilizing agent was carried out. The components inter-acted in a molar ratio ν(M):ν(Trp)=1:2. The concentrations in the resulting solution used were C(M)=10^−3^M (C_Ag_=C_Au_=0,5 10^−3^ M) and C(Trp)=2•10^−3^M. The molar ratio of Ag to Au was 1:1 for both cases.

The absorption spectra of the colloidal solutions of Ag/Au NPs were recorded in the UV-visible region with a spectrophotometer, Lambda 35 (Perkin-Elmer, United States), in 1 cm quartz cells. The particle size distribution function was studied by a laser correlation spectrometer (LCS) ZetaSizer-3 (Malvern Instruments, Worcestershire, United Kingdom) equipped with a correlator Multi8 (computing correlator type 7032 ce, Malvern Instruments, Worcester-shire, United Kingdom). The hydrodynamic diameter of the NPs was calculated from the diffusion coefficient by the Stokes-Einstein equation. 1 ml of studied suspension was placed in a cylindrical optical glass cell with a diameter of 10 mm, which was located in a thermostated sample holder of a laser correlation spectrometer. Registration and statistical processing of the scattered laser light at 90° from the suspension (helium-neon laser LGN–111 was used with power output of 25 mW and wavelength of 633 nm) was performed three times during 300 seconds. The resulting autocorrelation function was treated with standard computer programs PCS–Size mode v 1.61.

Electron microscopic images were obtained with a transmission electron microscope JEM–100C (JEOL, Japan) with an accelerating voltage of 100 kV. For the calculation of particle size distribution, the program ImageJ (National Institutes of Health (NIH), United States) was used.

### 2.2 Animal husbandry and the NPs' treatment

All the mice employed in our studies were congenic in the C57BL/6J genetic background and were treated and maintained according to the NIH Guide for the Care and Use of Laboratory Animals [7]. The mice of either sex, aged 12 weeks and weighing 18–22 g, were used in the experiments. Every five mice of the same sex were housed in cages containing a sterile paddy husk as bedding in a ventilated animal facility with a controlled temperature (21 ± 2 °C). All the mice were maintained on a laboratory complete diet with free access to water. Forty mice of each sex were randomly divided into five groups: one control group (intact animals) and four experimental groups with different NPs and vehicles respectively. The mice received intra-peritoneal (i.p.) injections of approximately 100 μL of the NPs solution (adjusting the final volume with the animal weight for the given dose) at a dose of 500 μg/kg/day daily for 12 days. The corresponding groups were administered with vehicle solutions (2•10^−3^ M tryptophan or 4,5•10^−3^ M sodium dodecyl sulphate). Intact animals of each sex (with no injections) were included as a control group. The body weight of the animals and their behaviour were carefully recorded, daily, during the course of the experiment. One day after the last injection (day 13), the mice were killed and the blood, liver, spleen, kidney, lungs and brain were collected and weighted immediately. Serum from the mouse blood was isolated by centrifugation at 1,500 g for 10 min. The dissected tissues were frozen in liquid N_2_ and stored continuously without thawing at −80 °C until analysis, and for the quantification of the gold/silver content in each tissue.

### 2.3 Anti-tumour activity assessment

As a model of malignant tumour growth a Lewis lung carcinoma was used. Tumour strain was kindly provided from the National Bank of Cell Lines from Human and Animal Tissues by R. E. Kavetsky Institute of Experimental Pathology, Oncology and Radiobiology, NAS of Ukraine. The transplantation was performed by intra-muscular injection into a femoral muscle of 0.2 ml 10% cell suspension in saline (3 × 10^6^ cells/ml). Starting from day five after tumour cell inoculation, the animals were divided into groups receiving daily an intra-peritoneal suspension of NPs (Ag/Au/SDS and Ag/Au/Trp) at the dose mentioned above. The animal morphological parameters (animal weight, primary tumour size) and survival rate were monitored daily. Euthanasia of the animals (6–7 animals per group) was performed on day 18 after Lewis carcinoma transplantation under light ether anaesthesia. Following sacrifice, the lungs were photographed and the number of metastatic nodules was counted. Anti-tumour activity was determined based on the parameters of tumour growth inhibition, the survival rate and the rate of lung metastatic infiltration.

### 2.4 Gold content determination in tissues

Analytical procedures for the determination of gold in tissues were performed exactly as described by others [8]. Briefly, tissue samples were dried completely in a clean oven at 60–70 °C. Each dried tissue was digested in an open atmosphere to white ash using 30% hydrogen peroxide (Merck, Germany) at 50–60 °C followed by digestion with 0.1 mL concentrated ultra-pure trace metal-free nitric acid (Merck, Germany). The digested white ash was dissolved in 0.25 M nitric acid. The recovery rate for elemental gold after the sample preparation determined by the standard additions method was more than 90%. The concentration of gold was measured in the samples using atomic absorption spectrophotometer S-115M1-PC (Ukraine), equipped by the microelement automatic calculation software LAB_AAS (Selmi, Ukraine). Each digested sample was further diluted suitably (1:2-1:50 v/v, depending on the tissue type and amount) using 0.25 M ultra-pure nitric acid prior to analysis. Sample atomization was performed in a flame of acetylene:air mixture. The lamp was operated at a specific absorption line of gold at 242.8 nm; a deuterium lamp was used for background correction. The elemental gold standard was used for all calibration curves.

### 2.5 Liver and kidney function tests

Alanine aminotransferase (ALT, EC 2.6.1.2) and gamma-glutamyl transpeptidase (GGT, EC 2.3.2.2) enzymatic activities were determined in mouse serum to assess liver injury using a colorimetric kit from Felisit Diagnostics (Ukraine), according to the manufacturer's protocols. Blood creatinine and urea levels were determined to assess renal filtration using an enzymatic kit from Felisit Diagnostics (Ukraine), according to the manufacturer's protocols.

### 2.6 Oxidative damage measurement

The oxidative modification of hepatic proteins was determined by the assessment of both the level of protein carbonylation, as described by others [9], and the level of protein sulfhydryl groups [10]. The measurement of protein carbonyls was performed spectrophotometrically (λ=370 nm) following their covalent reaction with 2,4-dinitrophenylhydrazine with the formation of a stable 2,4-dinitrophenyl hydrazone product. The detection of protein sulfhydryl groups involved specific reaction with Ellman's reagent (5,5′-dithiobis (2-nitrobenzoic acid, DTNB) with a subsequent absorbance reading at 412 nm. Lipid peroxidation in the liver was determined spectrophotometrically (λ=532 nm) by assessing the level of thiobarbituric acid-reactive substances (TBRAS), as described by others [11].

### 2.7 Statistical analysis

All the data are presented as means ± SD. Statistical comparisons were first analysed by a one-way ANOVA followed by multiple comparisons employing Tukey's HSD *post hoc* test. *P*-values of <0.05 were considered statistically significant.

## 3. Results and discussion

For the successful application of nanomaterials in bioscience, it is important to understand the biological fate and potential toxicity of NPs. Despite the widespread use of nano-sized gold and silver, relatively few studies have been undertaken to determine the cytotoxic effects of Ag and Au NPs exposure. Several methodological approaches have been proposed to be used in the reduction of NP toxicity, including their conjugation and functionalization [1]. In the current work, we developed bimetallic gold and silver NPs for biomedical application which were potentially less toxic than Ag/Au/SDS in relation to a mouse model system based on clinical biochemical parameters and oxidative damage products estimation. The bimetallic Ag/Au composition of the studied NPs was selected based on our previous studies [12]. The NPs structure, electronic and optical properties, chemical stability and their activity in various biological processes differ greatly compared to the properties inherent to the individual metals [1]. The transition from monometallic to bimetallic NPs is accompanied with the change of the surface plasmon absorption energy compared to the components of the bimetallic compositions. As such, we can combine the benefits of both gold and silver in one bimetallic NP. The mentioned characteristics are largely dependent on the method of synthesis and the ratio of components. In this study, we applied an essential amino acid tryptophan that can simultaneously act as a reducing and stabilizing agent of gold, and which has the ability to reduce the toxic effect of NPs on living cells (besides, it is easy to observe spectrally).

### 3.1 Characterization of NPs

The formation of particles is confirmed by the appearance of the characteristic colour of Ag/Au NPs solutions and surface plasmon resonance (SPR) bands in their optical spectra.

**Figure 1.**
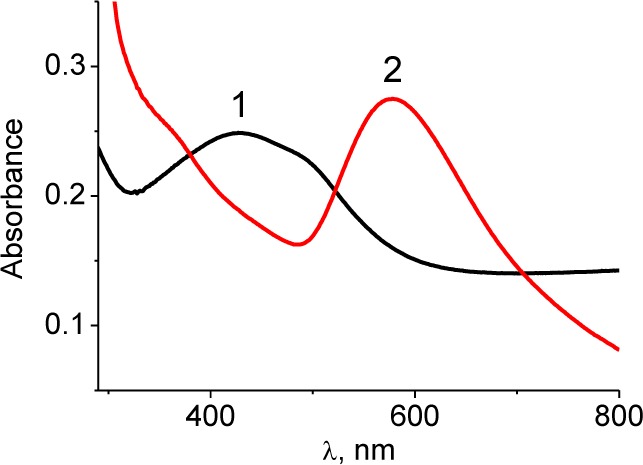
The absorption spectra of bimetallic NPs in solution. Line 1 corresponds to Ag/Au/SDS and line 2 to Ag/Au/Trp (dilution to metal concentration 10^−4^M)

The SPR maximum at 427 nm (Fig. 1) is typical for Ag/Au/SDS alloy NPs and has an intermediate position between the maxima of monometallic Ag and Au NPs at 400 and 520 nm, respectively.

**Figure 2.**
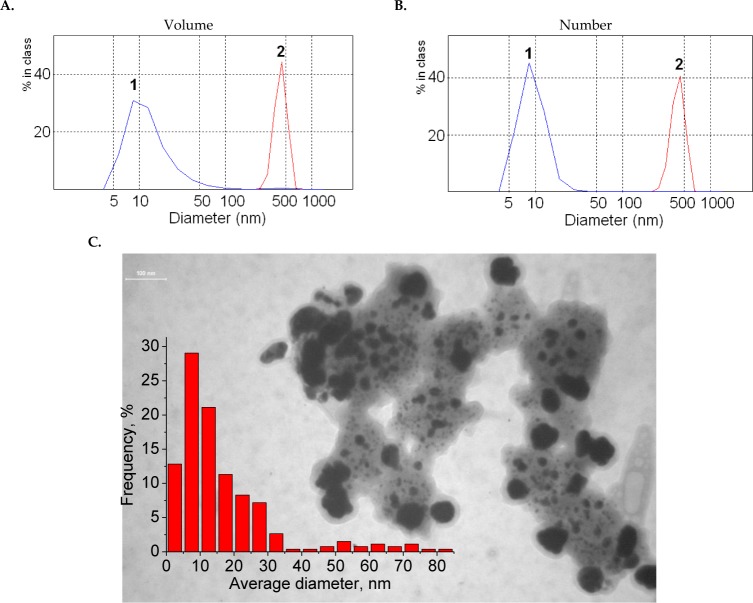
The size distribution of particles in colloids by volume and number according to LCS data (A., B.): Ag/Au/SDS (1) and Ag/Au/Trp (2). TEM image and size distribution of particles in Ag/Au/Trp colloids (C.)

The SPR maximum of AgAu/Trp is shifted to longer wavelengths (578 nm) compared to the absorption maximum of monometallic Au/Trp NPs (560 nm) and indicates the formation of large aggregates in the system. According to the data obtained with a laser correlation spectrometer (LCS) in the case of the simultaneous reduction of metals in two specified systems, the particles of different sizes were generated (Fig. 2). For the Ag/Au/SDS system, where the strong reductant as sodium tetrahydroborate is used and where there is a competition of nucleation and growth of nuclei processes with the domination of the first process over the second one, the particles of 8–12 nm are mainly formed. In the case of a weak reducing agent – the amino acid tryptophan – the possibility of multi-electron interactions between the components occurs due to the formation of charge transfer complexes between the tryptophan donor groups and the gold ions/NPs. As such, the reduction process significantly slows down, leading to the growth of large particles with an average size of 370–450 nm (Fig. 2B.). However, such a large size of several hundred nanometres is related not to the actual size of Ag/Au/Trp NPs but to their aggregates in a polymeric matrix arising from the oxidative polymerization of indole groups of tryptophan during reduction/stabilization. This is confirmed by the TEM image of the Ag/Au/Trp colloid (Fig. 2C.).

The large aggregates observed consist of a plurality of smaller particles, mainly about 5–15 nm, and a small number of particles of 50–70 nm. Thus, for both Ag/Au/SDS and Ag/Au/Trp systems in colloids, irrespective to the type and strength of the reducing agent, the NP with an average size of around 10 nm are formed.

### 3.2. Tryptophan-assisted synthesis reduces gold/silver NP potential hepato-and nephrotoxicity

In the present study, we explored the relationship of NP synthesis with their hepato-and nephrotoxicity *in vivo*. The NPs employed in this study had an average size of 10 nm (Fig. 1, Fig 2). The mice were treated with bimetallic Ag/AuNPs, prepared with a different stabilizing agent, by intra-peritoneal injection in the dose of 500 μg/kg/day for 12 days and daily examined for any changes in the morphology and behaviour. All the treated male and female mice survived throughout the experimental period without exhibiting any abnormalities. The mice did not show any pronounced symptoms of toxicity, such as weight loss, changes in food consumption or water intake, or changes in fur colour. The visual examination of NP-treated organs did not show any significant morphological changes in comparison to the control. The values of the visceral organ (liver, kidney, spleen) weights did not differ in either the male or female mice among the experimental and control groups (data not shown).

**Figure 3.**
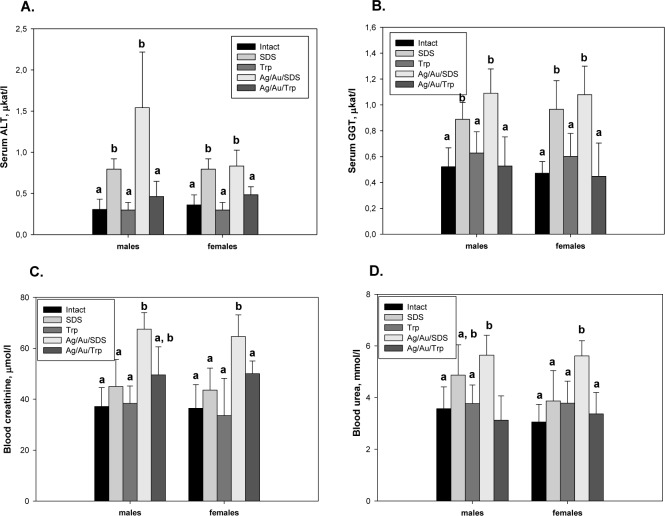
Liver injury and renal filtration capacity in mice after the daily intra-peritoneal administration of NPs (Ag/Au/SDS and Ag/Au/Trp) at a dose of 500 μg/kg/day for 12 days. Serum alanine aminotransferase (Panel A) and gamma-glutamyl transpeptidase activities (Panel B) in mice after NP administration. Blood creatinine (Panel C) and urea (Panel D) levels in mice after NP administration. All the values are given as the mean ± 1 S.D., n=7 for each group. Values marked with different letters (a, b, c) are statistically different, *P* < 0.05

More information was retrieved from biochemical parameters reflecting the functional state of the liver and kidneys. These parameters revealed the hidden NP toxicity, especially for those obtained using SDS as a stabilizer. ALT and GGT activities showed slight but significant elevation for both sexes under Ag/Au/SDS NP administration (Fig. 3A and 3B). The increased serum ALT and GGT activities were noted for both SDS and Ag/Au/SDS-treated groups, suggesting that the observed effect was instead caused by the stabilizer used in the synthesis rather than the metal component by itself. The most pronounced effect was observed in serum ALT activity in males, suggesting their greater susceptibility to the hepatotoxicity of Ag/Au/SDS NPs. This hepatotoxic effect was not observed for mice after Ag/Au/Trp administration (Fig. 3A and 3B).

The level of blood creatinine and urea symptomatic of the renal functions was also increased compared to the intact animals. However, the significant changes in values reflecting renal filtration capacity were observed only in groups of animals of both sexes treated with Ag/Au/SDS NPs. There were no significant changes in the blood creatinine and urea levels for mice treated with tryptophan-stabilized Ag/Au NPs in comparison to either tryptophan-treated or intact animals (Fig. 3C and 3D).

The method used in the current work involved tryptophan as a reducing/stabilizing agent, allowing the obtaining of NPs about the same size as those involving SDS. Thus, the type of stabilizing agent we consider to be a main feature underlying the observed reduction of the hepato-and nephrotoxicity of Ag/Au/Trp. Several studies confirmed the importance and the great influence of NP size on their biodistribution and interaction with complex, *in vivo*, whole-animal biological systems, where NPs of small sizes (with associated ultrahigh activity) were shown to interact with local tissues and provoke dysfunctions of the organs [13, 14]. The dependence on the size, shape and the possibility of internalization with corresponding cytological effects were shown for the functionalized gold NPs [15, 16]. An additional and often key factor that may influence the toxicity of NPs is the chemicals (or “initial reagents”) used during the synthesis; thus, in considering NPs as a multicomponent system the surface capping agents should be taken into account as permanent components of this system.

The origin of NPs' cytotoxicity from the surfactant/stabilizer used was already shown for Au NPs stabilized with cetyltrimethylammonium bromide [3, 17]. Thus, applying the protective agent that replaces potentially cytotoxic SDS [18] during the preparation of NPs may result in the observed lower *in vivo* toxicity.

Thus, in the present study an approach involving tryptophan as a stabilizer during Ag/Au NPs synthesis was used aiming to reduce the toxicity observed in mice. The performed biochemical studies revealed that liver and kidneys are target organs for the administered NPs, raising the possibility of their potential hepato-and nephrotoxicity. These were further demonstrated by the measurements of blood biochemical indexes (ALT, GGT, creatinine, urea), reflecting the renal and hepatic functions of the experimental mice. During the administration of Ag/Au tryptophan-stabilized NPs, the studied parameters did not differ from the intact animals, in contrast to that observed in mice continuously administered with NPs obtained by the classical protocol involving SDS, where an elevation in the serum ALT, GGT, creatinine and urea levels suggested the presence of marked hepatic and renal damage after continuous NP administration.

### 3.3 Tryptophan-assisted synthesis affects gold/silver NP biodistribution in vivo

**Figure 4.**
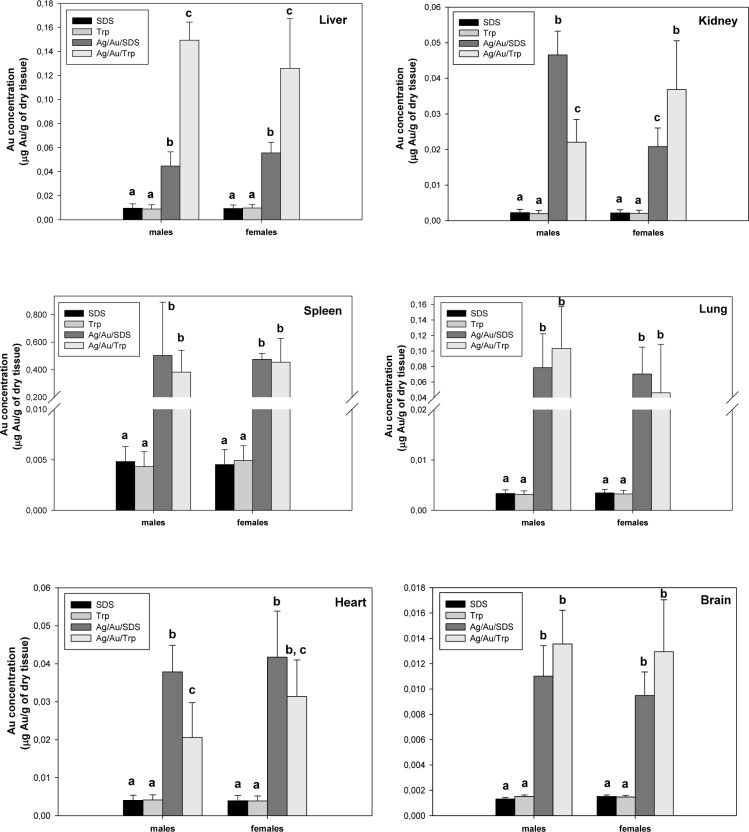
Gold element level in body tissues. The values correspond to the gold concentration relative to the weight of the dry tissue. All values are given as the mean ± 1 S.D., n=7 for each group and genotype. Values marked with different letters (a, b, c) are statistically different, *P* < 0.05

The distribution of the administered NPs was detected based on the gold element accumulation in diverse organs such as the liver, spleen, kidneys, lungs, brain and heart. The NPs were distributed in all the organs, with the highest accumulation in the spleen, followed by the liver, lungs, heart, kidneys and brain (Fig. 4). It is known that nanoscale particles as any inorganic compound are captured by the cells of the reticuloendothelial system (RES); therefore, they are predominantly accumulated in the spleen and liver in relatively high concentrations [19]. There were no significant differences in the Ag/Au/SDS NPs' accumulation in different-sex animals, except for the kidneys, where the level of the gold element was observed twice as much in males compared to females. Using tryptophan for the stabilization of the NPs affected their biodistribution within the body, reflected in the changes of the gold element level in the liver, kidneys and heart. There was a statistically significant increase in the gold concentration of hepatic tissue samples from the groups exposed to Ag/Au/Trp NPs in this study. The difference observed for the liver, where there was an almost three-fold increase in the accumulation of Ag/Au/Trp NPs compared to Ag/Au/SDS, may be explained by the fact that the liver is considered as a target organ for silver and gold NPs, which are taken up by the cells of RES [5, 14]. Being captured, the NPs were observed as being located in the cytoplasmic vesicles and lysosomes of liver-resident macrophages – namely, Kupffer cells [19].

In addition, the lower level of gold in the heart of males and females (for 45% and 25% respectively) was noted in groups receiving Ag/Au/Trp NPs compared to SDS-stabilized colloids. An opposite tendency was observed in the tryptophan-stabilized NP distribution in the kidneys of mice, where the amount of gold determined in the male kidney was significantly lower (although higher in the female kidney) compared to the sex groups treated with Ag/Au/SDS (Fig. 4), indicating a marked gender-dependent distribution of this NP type with respect to the kidneys. Our results are consistent with the published data on NP distribution in animals of different sexes [20, 21].

### 3.4 Tryptophan-stabilized Ag/Au NPs exhibit lower pro-oxidant activity in relation to cellular lipids, but do not differ from the SDS-stabilized NPs in their ability to induce protein oxidation

The penetrating of NPs into the cell through endocytosis can disrupt the integrity of plasma and/or intracellular membranes [17], leading to cell death in high local concentrations. Earlier reports have emphasized the role played by oxidative stress in NP toxicity [22-25]. Oxidative stress has specific effects in the cells, including oxidative damage to lipids and proteins.

The performed analysis showed a significant increase in thiobarbituric acid reactive substances' formation in the visceral organs of animals treated with Ag/Au/SDS NPs. (Table 1.).

**Table 1.** Oxidative damage products in mouse liver, kidneys and spleen after daily intra-peritoneal administration of NPs (Ag/Au/SDS and Ag/Au/Trp) at a dose of 500 μg/kg/day for 12 days. All values are given as the mean ± 1 S.D., n=6 for each group. Values marked with different letters (a, b, c) are statistically different, *P* < 0.05

Organ	Group	TBRAS, nmol/mg protein		Protein carbonyl derivatives, μmol/mg protein		Protein thiol groups, μmol/mg protein	

		males	females	males	females	males	females
	Intact	27,77±3,50^a^	29,31±3,24^a^	0,27±0,06^a^	0,27±0,09^a^	13,65±1,88^a^	13,22±1,82^a^
**Liver**	Ag/Au/SDS	74,95±17,96^b^	74,66±18,20^b^	0,97±0,22^b^	1,16±0,34^b^	4,39±1,78^b^	4,19±1,17^b^
	Ag/Au/Trp	39,36±9,81^a^	28,81±15,34^a^	0,76±0,15^b^	0,69±0,22^b^	9,03±3,70^c^	9,01±2,33^c^

	Intact	38,29±2,90^a^	38,70±3,46^a^	0,45±0,10^a^	0,45±0,08^a^	7,34±0,90^a^	7,20±1,64^a^
**Kidney**	Ag/Au/SDS	81,91±21,77^b^	33,59±15,41^a^	0,97±0,26^b^	1,15±0,35^b^	4,39±1,23^b^	4,16±2,64^b^
	Ag/Au/Trp	34,57±12,79^a^	79,33±23,42^b^	0,83±0,21^b^	0,83±0,17^b^	5,56±1,97^a,b^	5,11±1,88^a,b^

	Intact	20,91±2,20^a^	29,13±2,86^b^	0,40±0,05^a^	0,39±0,07^a^	8,53±0,91^a^	8,24±1,32^a^
**Spleen**	Ag/Au/SDS	97,64±18,06^c^	66,85±14,88^c^	0,67±0,07^b^	0,87±0,13^b^	5,78±2,45^b^	5,49±2,12^b^
	Ag/Au/Trp	30,75±11,43^b^	27,68±13,95^b^	0,61±0,13^b^	0,49±0,17^c^	5,37±2,41^b^	5,73±2,52^b^

The intra-peritoneal administration of NPs during 12 days in daily doses of 500 μg/kg/day led to the development of enhanced lipid peroxidation as evidenced by an increase of TBARS by 2.7 in the liver, 2.1 in the kidneys and almost 4.5 in the spleen (Table 1.). However, such prominent features of lipid peroxidation were not observed in the liver of Ag/Au/Trp NP-administered animals. The values of the hepatic TBARS level in these groups did not exceed the parameters of the intact mice of both sexes. A significant increase in the TBARS was found in female kidneys after the Ag/Au/Trp NPs' treatment, reflecting the tendency observed for the accumulation of this NP type in mouse kidneys. The female mice exhibited near-normal levels of lipid peroxidation products in the kidney when administered with SDS-stabilized NPs; however, in males this was observed during tryptophan-stabilized NPs treatment. The analysis of the accumulation of protein oxidative modifications (i.e., carbonyl derivatives and the level of sulfhydryl groups) showed that the NPs used, irrespective to the stabilizer used, are able to cause oxidative degradation of the cellular proteins (Table 1.). Moreover, the relative values of the carbonyl derivatives' accumulation observed in the liver, kidneys and spleen showed higher pro-oxidant activity for the SDS-stabilized NPs. Presumably, during the penetration of NPs into the cell, the loss of the surface-associated stabilizer occurs and its role in the reduction of the pro-oxidant activity is neglected. This may explain the same accumulation values of the protein oxidation products regardless of the type of stabilizer used.

### 3.5 Tryptophan-stabilized Ag/Au NPs possess anti-tumour activity

**Figure 5.**
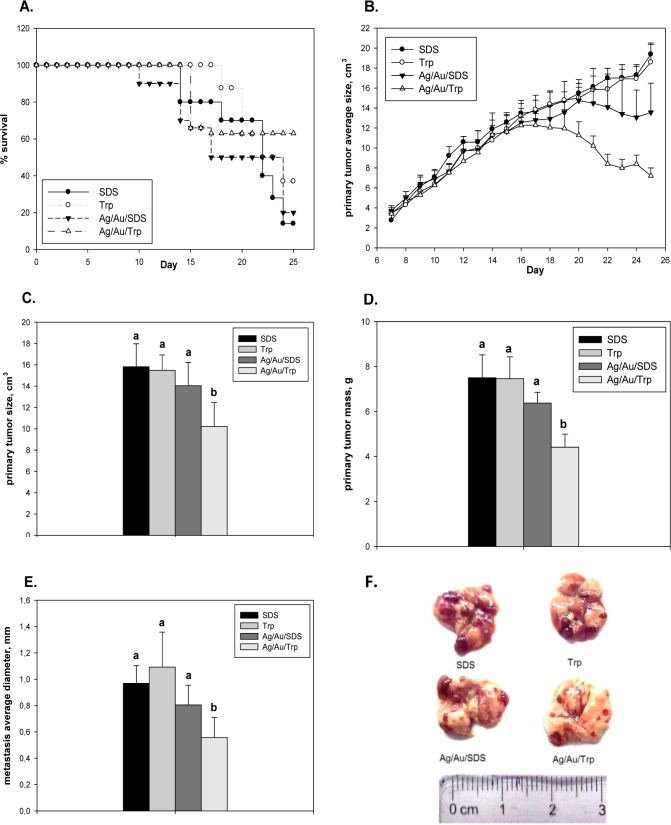
Anti-tumour activity of NPs. Survival rate (Panel A) and primary tumour size (cm^3^) (Panel B) in mice of experimental and control groups at different experimental periods during Lewis lung carcinoma growth; primary tumour size (cm^3^) (Panel C) and mass (g) (Panel D) on day 18 of Lewis lung carcinoma growth; metastasis average diameter (mm) (Panel E) and representative photograph (Panel F) in lungs at day 18 of Lewis lung carcinoma growth. All values are given as the mean ± 1 S.D., n=7 for each group and genotype. Values marked with different letters (a, b) are statistically different, *P* < 0.05

The studies showed that the daily intra-peritoneal administration of a bimetallic nanocomposite at a dose of 500 μg/kg led to the inhibition of the growth of the Lewis lung carcinoma primary tumour from day 16 of the experiment (day 11 of NP administration). However, the likely positive result was detected only in the group of animals with a carcinoma treated with Ag/Au/Trp (Fig. 5B). On the 21^st^ day of the experiment, the rate of tumour growth inhibition (by weight) was 41% in this group compared to 15% in the group receiving Ag/Au/SDS, while the observed decrease in the size was 34% for Ag/Au/Trp-treated animals and only 11% for animals treated with Ag/Au/SDS. On the 25^th^ day of the experiment, the inhibition of the growth of the Lewis carcinoma primary tumour accounted for 61% compared to 30% in the animals injected with Ag/Au/SDS (Fig. 5B,5C, 5D) after 20 days of Ag/Au/Trp administration. Along with less anti-tumour activity, the Ag/Au/SDS nanocomposite had higher toxicity (Fig. 5A). This was evidenced by higher levels of mortality in animals receiving a daily dose of 500 μg/kg of this nanocomposite. In particular, after a seven day period of the administration of NPs, the mortality rate was 35%, reaching 50% on day 14 of administration (day 21 of the experiment) and was 85% at the terminal stages of observation. However, in the Ag/Au/Trp-treated group, the level of mortality did not exceed 40% throughout the experimental period (Fig. 5A). During the administration of the bimetallic nanocomposite, the frequency of lung metastasis did not differ among the groups, and was 100%. However, after two weeks of NP injections at a dose of 500 μg/kg, the metastasis inhibition index was 26% for the Ag/Au/SDS-treated mice and 43% for the Ag/Au/Trp (Fig. 5E). The severity assessment of the of metastatic process revealed that the use of the studied 5F). The differences in the number of metastases and their diameter in the group Ag/Au/SDS were not statistically significant and had the character of a trend, while after the administration of bimetallic Ag/Au/Trp NPs the average diameter of the metastasis at 21 days was statistically significantly lower compared with the control group (Fig. 5E). Only metastases with a small diameter were identified in the lungs of the Ag/Au/Trp-treated animals; moreover, the number of metastases was also much lower than in control and Ag/Au/SDS-treated mice.

In summary the data generated from the present study supports the fact that the use of tryptophan in NP synthesis is effective in attenuating the potential hepatotoxicity and nephrotoxicity of NPs during their *in vivo* application.
